# Visual learning performance in free-flying honey bees is independent of sucrose and light responsiveness and depends on training context

**DOI:** 10.1038/s41598-025-34900-9

**Published:** 2026-01-09

**Authors:** Valerie Kuklovsky, Aurore Avarguès-Weber, Martin Giurfa, Ricarda Scheiner

**Affiliations:** 1 Faculty of Biology, Behavioral Physiology and Sociobiology, Biocenter, Julius-Maximilians-Universität Würzburg, 97074 Würzburg, Germany; 2 Centre de Recherches sur la Cognition Animale (CRCA), Centre de Biologie Intégrative (CBI), CNRS, UPS, Université de Toulouse, 31400 Toulouse, France; 3 CNRS, Inserm, Neuro-SU, Sorbonne Université, 75005 Paris, France; 4 CNRS, Inserm, Institut de Biologie Paris-Seine, IBPS, Sorbonne Université, 75005 Paris, France; 5 Present Address: Department of Biology and Center for the Advanced Study of Collective Behaviour, University of Konstanz, 78464 Konstanz, Germany

**Keywords:** Honey bee, Configural learning, Elemental learning, Negative patterning, Non-elemental learning, Phototaxis, Reversal learning, Stimulus responsiveness, Sucrose responsiveness, Ecology, Ecology, Neuroscience, Zoology

## Abstract

Associative learning plays a fundamental role in the life of honey bees, especially in the context of foraging for food sources. This learning capacity can be investigated through controlled experiments conducted under laboratory, semi-natural, and near-natural conditions, to understand the general principles of learning and motivation. Honey bees can be trained to solve different elemental and non-elemental learning tasks by pairing a conditioned stimulus such as an odor with sucrose as an unconditioned stimulus and reward. Laboratory studies with restrained bees demonstrated that sucrose responsiveness is positively correlated with both elemental olfactory learning performance and responsiveness to stimuli of different sensory modalities, such as odors and visual stimuli. Here, we tested for the first time how responsiveness to sucrose and light is related to performance in elemental and non-elemental visual learning under free-flying conditions. Sensory responsiveness and learning proficiency did not correlate, nor did sucrose responsiveness correlate with responsiveness to light. These results indicate that relationships among responsiveness to sucrose and light and learning proficiency, as established under restrained laboratory conditions, may not translate to the natural behavior of bees in the field. This finding points toward the context-dependent importance of responsiveness to light and sucrose during associative learning under restrained or free-flying conditions.

**Supplementary Information:**

The online version contains supplementary material available at 10.1038/s41598-025-34900-9.

## Introduction

Associative learning is the process by which information about predictive relationships between concurrent events and stimuli in the environment is acquired^1^. This capacity is widespread among animal taxa and is a fundamental component of adaptive behavior, enabling an organism to anticipate one event on the basis of the presence of another^2^. Two main categories of associative learning can be distinguished: (1) Pavlovian conditioning, also known as classical conditioning^3^, involves an initially neutral stimulus (conditioned stimulus, CS) becoming associated with a stimulus that is biologically relevant to the animal (unconditioned stimulus, US). In Ivan Pavlov’s classic experiment, food (the US), which naturally elicits salivation in dogs (the unconditioned response, UR), was paired with the sound of a bell (the CS). After learning this CS‒US association, dogs in response to the sound alone, which became the conditioned response (CR)^3^. (2) Operant conditioning involves learning to associate one’s own behavior, such as pressing a lever, with a reinforcing outcome, such as receiving a food reward^4^. Both types of conditioning can involve either aversive or appetitive reinforcement and can vary in complexity depending on whether unambiguous (elemental learning) or ambiguous (non-elemental learning) relationships are established between stimuli.

Proficiency in associative learning is influenced by numerous factors, among which the perception of the CS and the US in Pavlovian conditioning or the discriminative stimuli and reinforcement in operant conditioning play a major role^5–7^. In this context, more salient stimuli—that is, those that are more noticeable or prominent owing to their physical intensity, perceptual distinctiveness, or relevance to the animal’s current needs are learned more readily than less salient ones^8^. Similarly, the strength of the US or reinforcement i.e. the reward value, has a profound effect on associative strength and learning outcomes^9,10^. Both the reward value and the salience of the CS or discriminative stimuli depend heavily on how the sensory systems detect and process their properties^11^. For example, an animal with heightened sensory responsiveness to a food reward is likely to learn better than one with lower sensory responsiveness. Understanding how and to what extent stimulus salience and reward value contribute to performance in associative learning tasks is a key topic in the study of learning, memory and cognition across humans and non-human animal taxa.

The honey bee (*Apis mellifera*) is a long-standing model organism for the study of learning, memory and cognition^12–16^. It is particularly well suited for investigating the relationship between sensory responsiveness and associative learning performance. First, the established protocols enable both Pavlovian and operant conditioning experiments with individual bees. In Pavlovian conditioning, restrained bees are trained and tested in a controlled laboratory environment^17^. Sucrose solution serves as the US, eliciting the extension of the proboscis when the bees’ antennae are stimulated—a response known as the proboscis extension response (PER). An odorant, which does not elicit an innate response, is used as the CS and is presented in close temporal association with the sucrose reward^18^. These experiments offer the advantages of constant external conditions and precise control of CS and US presentations, ensuring consistent stimuli for all tested bees. Operant conditioning protocols typically involve free-flying, color-marked bees trained individually in a more naturalistic setup^13,19,20^. In these experiments, a bee is displaced by the experimenter to a test site and rewarded with sucrose solution at the new location. Once the bee returns regularly, training stimuli are introduced, and the correct target is associated with a sucrose reward. These stimuli may include odorants, colors, shapes and patterns, among others^13,20–25^. Although primarily operant, this protocol also allows for Pavlovian associations between the stimuli and reward^26,27^, mirroring the natural nectar foraging behavior of bees^28^.

Second, well-established protocols exist that reliably assess an individual bee’s responsiveness to various stimuli. The PER assay is commonly used to measure sucrose responsiveness before Pavlovian conditioning using sucrose as US^29–33^. In this assay, the antennae of a restrained bee are stimulated with ascending sucrose concentrations to determine the concentrations at which bees exhibit PER. Bees that respond even to low concentrations are considered highly responsive to sucrose, whereas others require relatively high concentrations to elicit PER^34^. Higher sucrose responsiveness has been linked to better associative learning performance^29–33,35,36^. Additionally, a phototaxis assay is regularly used to assess the responsiveness of bees to light. In this assay, a bee is placed in a dark arena and exposed to colored light stimuli of increasing light intensities^18,37–40^. The time it takes for a bee to reach the light stimulus serves as a proxy for its responsiveness to light, with shorter walking times indicating higher sensitivity. Interestingly, sucrose responsiveness is positively correlated with responsiveness to light^37,41,42^ as well as to other stimuli, such as odors and pollen^43^.

However, whether sucrose responsiveness is also positively correlated with operant learning performance under free-flying conditions remained an unresolved question. In addition to a learning context, Mujagic and Erber^44^ first measured the sucrose acceptance of free-flying bees at an artificial feeder and then harnessed the same bees to assess their sucrose responsiveness in the laboratory via the PER assay. They reported no correlation between sucrose acceptance in the field and responsiveness in the laboratory. This finding led to the conclusion that freedom of movement and thus active sensing may play a critical role in modulating sucrose responsiveness^44^. Similar results have been observed in conditioning experiments: when bees were first conditioned in a set-up that allowed free movement and active sensing and then transferred to a harnessing set-up to perform the same task, they were unable to transfer their learned choice. This phenomenon has been demonstrated in both visual^45^ and olfactory conditioning^46^, underscoring the decisive role of active sensing in learning experiments.

The main goal of this study was to investigate whether the predictive relationship between sensory responsiveness (to sucrose and light, respectively) and learning performance, previously demonstrated in laboratory experiments with restrained bees, also applies to free-flying bees in the field. To overcome the challenge of testing responsiveness under restrained conditions before subjecting bees to free-flying experiments, we reversed the experimental sequence, first testing the bees in free-flight tasks and subsequently quantifying their sensory responsiveness. Moreover, we investigated whether the positive associations between sucrose/light responsiveness and learning performance depend on task complexity. To this end, we replicated a previous experiment^22^ in which free-flying bees were sequentially trained on a visual reversal learning task followed by a negative patterning task, allowing us to correlate individual performances across these tasks. Both tasks are cases of non-elemental associative learning: reversal learning introduces temporal ambiguity in the association, requesting cognitive flexibility, whereas the negative patterning task relies on the ability for configural processing (processing a compound differently as the sum of its parts). We hypothesized that sucrose responsiveness could account for a proportion of the inter-individual variability observed in learning proficiency during reversal learning and negative patterning tasks. Furthermore, we predicted that the effect of sucrose responsiveness would decrease as task complexity increased. Indeed, success in non-elemental learning tasks depends on increased cognitive functions, which may be less directly dependent on appetitive motivation.

## Results

### Individual consistency in learning performance

We tested all of the bees across two consecutive learning tasks: reversal learning (RL), which included a first phase followed by a reversal of stimulus contingencies in the second phase, and negative patterning, in which two single stimuli were rewarded while their combination was not (NP). This approach aimed to investigate whether proficiency in solving one task correlates with the ability to learn a different task. Only bees which successfully learned in the first phase of reversal learning (*n* = *26*, defined as ≥ 60% correct choices in the non-reinforced tests) were included in correlations involving the second phase of reversal learning. The order in which the learning tasks were conducted, i.e. either reversal learning or negative patterning was performed first, had no effect on test performance (GLMM*; order: n* = *30; 1*^*st*^* phase RL:* ${\chi }_{(1)}^{ 2}$= *0.89, p* = *0.35; 2*^*nd*^* phase RL:* ${\chi }_{(1)}^{ 2}$ = *1.07, p* = *0.30; NP:* ${\chi }_{(1)}^{ 2}$ = *0.29, p* = *0.59).* Similarly, the identity of the color rewarded first in reversal learning (yellow or greenish yellow) did not influence test performance (GLMM*; group_RL: n* = *30, 1*^*st*^* phase RL:* ${\chi }_{(1)}^{ 2}$= *0.11, p* = *0.74, 2*^*nd*^* phase RL:* ${\chi }_{(1)}^{ 2}$ = *2.93, p* = *0.09, NP:* ${\chi }_{(1)}^{ 2}$ = *0.28, p* = *0.59).* Therefore, the data were pooled for subsequent statistical analyses.

**Fig. 1:**
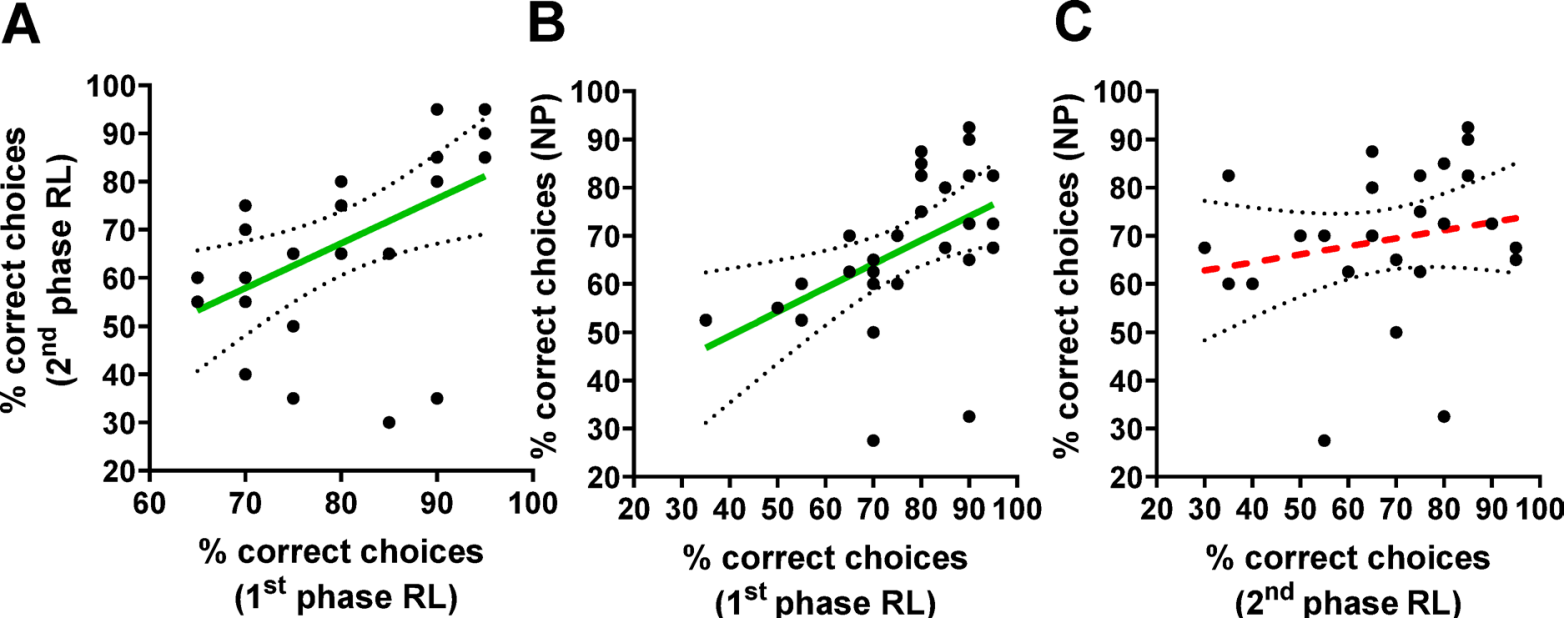
Individual consistency in the test performances of the first and second phases of reversal learning (1^st^ phase and 2^nd^ phase RL) and negative patterning (NP). The graphs show the Spearman rank correlations of individual test performance measured as the percentage of correct choices made during the 20-choice non-reinforced tests for each learning task. For all correlations involving the 2^nd^ phase of reversal learning, only bees (*n* = *26*) that successfully learned the 1^st^ phase (≥ 60% correct choices in the test) were included. This criterion was necessary, as reversal learning ability can be assessed only in individuals who have acquired the initial A+ versus B− discrimination of the 1st phase. The individual test performances were significantly and positively correlated between (**A**) the 1^st^ and 2^nd^ phases of reversal learning (*n* = *26, rho* = *0.62, p* = *0.0008, R*^*2*^ = *0.26*) and (**B**) between the 1^st^ phase of reversal learning and negative patterning. (*n* = *30, rho* = *0.61, p* = *0.0003, R*^*2*^ = *0.22*). (**C**) A positive, albeit non-significant, correlation was observed between individual test performance in the second phase of reversal learning and negative patterning (*n* = *26, rho* = *0.33, p* = *0.1, R*^*2*^ = *0.04*). Each dot represents data from a single bee. The solid green lines indicate significant positive correlations, whereas the red dotted lines represent non-significant positive correlations; 95% confidence intervals for linear regressions are shown as gray dotted lines.

Individual test performance in the first phase of reversal learning was positively correlated with that in the second phase (Spearman rank correlation, *n* = *26, rho* = *0.62, p* = *0.0008;* Fig. 1A). In other words, stronger learning performance in the first phase corresponded to better learning performance in the second phase. Additionally, we found a significant positive correlation between test performance in the first phase of reversal learning and negative patterning (*n* = *30, rho* = *0.61, p* = *0.0003;* Fig. 1B). This finding indicates that better elemental learning in the first phase of reversal learning was associated with stronger non-elemental learning in the negative patterning task. Finally, the correlation between individual test performance in the second phase of reversal learning and negative patterning showed a non-significant positive trend (*n* = *26, rho* = *0.33, p* = *0.1; *Fig. 1C). This pattern of correlations is consistent with earlier findings^22^.

## Relationships among sucrose responsiveness, responsiveness to light and visual learning performance in free-flying bees

### Sucrose responsiveness

Following the completion of the learning tasks, the sucrose responsiveness of the bees was tested. Overall, the bees exhibited high responsiveness, with a median gustatory responsiveness score (GRS) of 6, indicating that they responded to nearly all of the sucrose concentrations tested. Nevertheless, PER probability increased significantly across sucrose concentrations (Friedman test, *n* = *29,* ${\chi }_{(6)}^{ 2}$ = *30.33, p* < *0.0001*), confirming graded gustatory responsiveness (see Fig. S3A in the supplementary material).

Comparisons with untrained control bees collected at a feeder either at the start (“control 1”) or at the end of the experiment (“control 2”) revealed no significant differences in GRS between trained and control bees. Specifically, GRSs did not differ between control 1 bees and trained bees (two-way Mann‒Whitney U test on GRSs; *n*_*untrained*_ = *19, n*_*trained*_ = *29, U* = *189, p* = *0.06*) or between control 2 bees and trained bees (*n*_*untrained*_ = *30, n*_*trained*_ = *29, U* = *374, p* = *0.35*; Fig. S2B). These findings suggest that the participation of free-flying bees in extensive visual learning protocols does not significantly alter sucrose responsiveness.

**Fig. 2:**
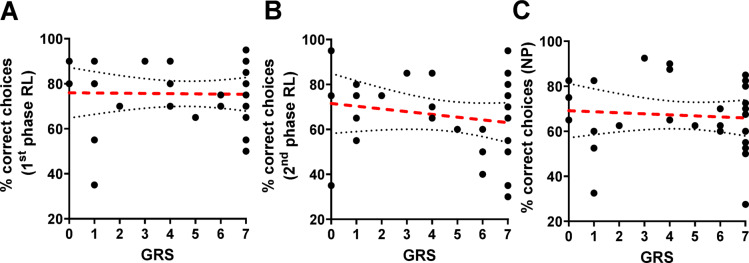
Relationships between gustatory response scores (GRS) of bees and their test performance in reversal learning and negative patterning. Test performance was assessed as the percentage of correct choices made during 20 decisions in the non-reinfored test of each learning task. No significant correlation was found between GRS and test performance in (**A**) the 1^st^ phase of reversal learning (*n* = *29, rho* = *− 0.08, p* = *0.68, R*^*2*^ = *0.0003*), (**B**) the 2^nd^ phase of reversal learning (*n* = *29, rho* = *− 0.15, p* = *0.43, R*^*2*^ = *0.04*) or (**C**) negative patterning (*n* = *29, rho* = *− 0.12, p* = *0.55, R*^*2*^ = *0.006*). Each dot represents data from a single bee. The regression red dashed lines represent nonsignificant correlations and are displayed alongside their 95% confidence intervals shown as gray dotted lines.

We next examined whether sucrose responsiveness reliably predicts visual learning performance, as it does for olfactory learning in harnessed bees. No significant correlation was found between the gustatory response score (GRS) and test performance in any of the learning tasks examined. Specifically, the GRS did not correlate with performance in the first phase of reversal learning (Spearman rank correlation; *n* = *29, rho* = *-0.08, p* = *0.68*; Fig. 2A), the second phase of reversal learning (*rho* = *0.11, p* = *0.55*; Fig. 2B)*,* or negative patterning (*rho* = *-0.11, p* = *0.55*; Fig. 2C). Additionally, we found no significant effect of the GRS on performance in the non-reinforced test of the 1^st^ phase of reversal learning and negative patterning (GLMM*; GRS: n* = *29, 1*^*st*^* phase RL:* ${\chi }_{(1)}^{ 2}$= *0.43, p* = *0.51; NP:* ${\chi }_{(1)}^{ 2}$ = *0.32, p* = *0.75).* However, we found a barely significant effect of the GRS on performance in the non-reinforced test of the second phase of reversal learning (GLMM*; GRS: n* = *29, 2*^*nd*^* phase RL:* ${\chi }_{(1)}^{ 2}$= *-2.00, p* = *0.05)*. Accordingly, bees with a high GRS perform worse in the second phase of reversal than do bees with a lower GRS. Notably, this slightly significant effect might be due to the relatively high number of low-performing bees with GRSs of 6 and 7, which occurs exclusively in the 2^nd^ phase of reversal learning but not in the first phase of reversal learning or in negative patterning (see Fig. 2). Taken together, these results indicate that sucrose responsiveness does not reliably predict visual learning performance in free-flying bees.

### Responsiveness to light

**Fig. 3:**
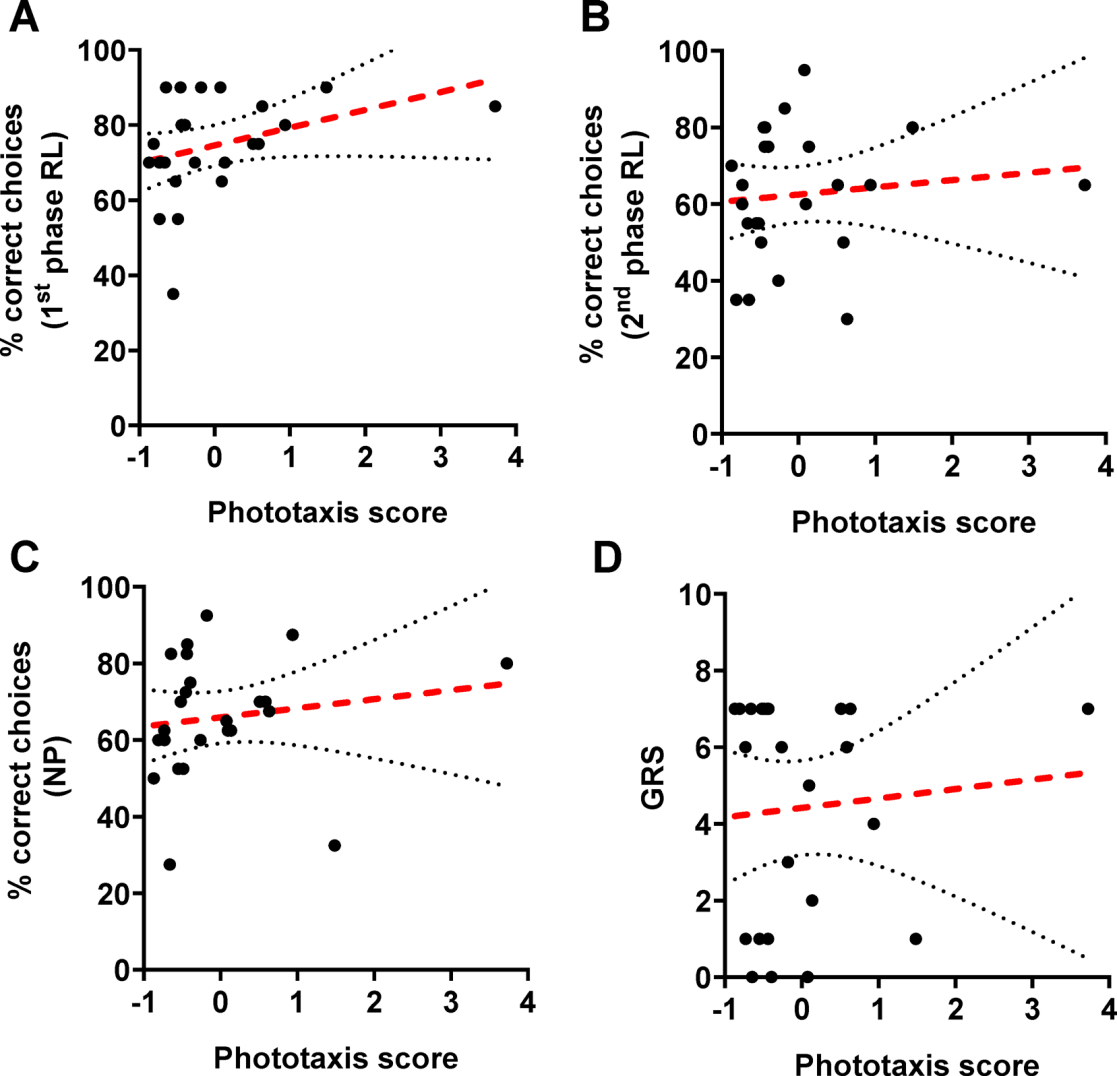
Relationships between learning performance and responsiveness to sucrose and light. Learning performance was assessed as the percentage of correct choices made in the non-reinforced test of each task. Sucrose responsiveness was quantified via the gustatory response score (GRS), which represents the number of proboscis extensions in response to antennal stimulation with water and six ascending sucrose concentrations. Responsiveness to light was quantified as the mean walking time (in seconds) to each of the six different relative light intensities. The composite phototaxis score was calculated from the six mean walking times via principal component analysis. No significant correlations were found between learning performance and the phototaxis score (*n* = *24*): (**A**) 1^st^ phase of reversal learning (1^st^ phase RL): *rho* = *0.35, p* = *0.09, R*^*2*^ = *0.12;* (**B**) 2^nd^ phase of reversal learning (2^nd^ phase RL): *rho* = *0.22, p* = *0.30, R*^*2*^ = *0.01;* (**C**) Negative patterning (NP): *rho* = *0.35, p* = *0.09, R*^*2*^ = *0.02.* (**D**) No significant correlation was found between the GRS and the phototaxis score (*rho* = *-0.09, p* = *0.67, R*^*2*^ = *0.007).* Each dot represents the data from a single bee. The regression red dashed lines represent nonsignificant correlations and are displayed alongside their 95% confidence intervals shown as gray dotted lines.

After evaluating sucrose responsiveness following visual conditioning in reversal learning and negative patterning, we next assessed responsiveness to light. Walking time in the arena used to test responsiveness to light significantly decreased with increasing light intensity (Spearman rank correlation; *n* = *24*, *rho* = *-0.35, p* < *0.0001;* Fig. S3), indicating stronger phototaxis to higher light intensities. Despite this, test performances in the first and second phases of reversal learning and in negative patterning did not correlate with phototactic scores (Spearman rank correlation, *n* = *24; 1*^*st*^* phase RL: rho* = *0.35, p* = *0.09; 2*^*nd*^* phase RL: rho* = *0.22; NP: rho* = *0.35, p* = *0.09;* Fig. 3A‒C). Furthermore, we found no significant effect of the mean walking time at the different light intensities on any of the learning tasks we tested (GLMM*; mean_WT: n* = *24, 1*^*st*^* phase RL:* ${\chi }_{(1)}^{ 2}$= *0.49, p* = *0.62; 2*^*nd*^* phase RL:* ${\chi }_{(1)}^{ 2}$ = *0.27, p* = *0.79; NP:* ${\chi }_{(1)}^{ 2}$ = *0.17, p* = *0.86).* Locomotor activity, measured as average walking speed, was not significantly correlated with test performance in any of the three learning tasks (Spearman rank correlation; *n* = *29*; 1^st^ phase RL: *rho* = *0.08, p* = *0.69*; 2^nd^ phase RL: *rho* = *0.02, p* = *0.92*; negative patterning: *rho* = *0.114, p* = *0.49*; Fig. S4).

### Relationship between sucrose responsiveness and responsiveness to light

Previous studies on harnessed bees reported a significant correlation between sucrose responsiveness and responsiveness to light^37,41,42^. To investigate whether the same relationship is present in free-flying bees, we analyzed the correlation between GRS and the phototaxis score. Our results revealed no significant correlation between the GRS and the phototaxis score (Spearman rank correlation, *n* = *24; rho* = *− 0.09, p* = *0.67;* Fig. 3D). These findings suggest that sucrose responsiveness does not predict responsiveness to light in free-flying bees.

## Discussion

In this study, we examined the relationships between sensory responsiveness (to sucrose and to light) and proficiency in visual learning tasks of different complexities in free-flying bees. Our goal was to better understand the factors underlying consistent individual differences in the cognitive abilities of honey bees. In the present study, we replicated the visual learning experiments described in^22^; free-flying honey bees were sequentially tested via visual reversal learning and negative patterning. This approach allowed us to assess bees’ proficiency in both elemental learning (1^st^ phase RL) and non-elemental learning (2^nd^ phase RL and NP). We then measured the responsiveness of these bees to sucrose and light under laboratory conditions. Correlational analyses were conducted to evaluate individual performance across the three learning tasks. Our results confirmed our prior findings^22^, namely, a positive correlation between individual performance in the first and second phase of reversal learning and between the first phase of reversal learning and negative patterning, whereas no significant correlation emerged between the second phase of reversal learning and negative patterning. These results reinforce our earlier findings and support the hypothesis that this specific pattern of correlations represents a distinct feature of the cognitive profile of honey bees, with distinct cognitive modules supporting flexibility and configural competences.

In PER conditioning studies with harnessed bees, sucrose responsiveness, measured prior to conditioning, has been shown to strongly correlate with performance in olfactory elemental learning tasks^29–33,35,36^. Here, we investigated whether a similar predictive relationship exists between responsiveness to light, sucrose responsiveness and visual learning proficiency in free-flying bees. Our results revealed no significant correlations between sucrose responsiveness or responsiveness to light and test performance across the three tasks.

There are, however, important differences between these studies and the present work. Notably, we did not assess responsiveness to sucrose or light in the same bees *prior* to conditioning, as was done with harnessed bees. This decision was based on the substantial negative impact of the harnessing procedure on the subsequent motivational state of free-flying bees, which would likely prevent them from returning to the experimental site for subsequent visual learning tasks. To overcome this problem, we first conditioned the bees in the visual learning task, then measured the GRS and subsequently assessed the mean walking times to different light intensities. We first harnessed the bees to test the GRS and then removed bees from the harness to perform the light responsiveness assay similar to^37^. This was done as we had to feed the bees with a controlled volume of sucrose solution after bringing them to the lab which is facilitated by harnessed conditions to ensure equivalent and sufficient level of satiety for all bees before both lab tests.

Another methodological difference is the use of 50% sucrose solution as unconditioned stimulus in our learning experiments while a 30% sucrose solution was used in the PER studies^29–33,35,36^. This difference in reward concentration reflects the distinct motivational demands of restrained versus free-flying paradigms. In PER studies, a sucrose concentration of 30% is sufficient to elicit PER in harnessed bees and aligns with the graded sucrose responsiveness assay. In contrast, free-flying bees require higher reward salience to ensure consistent foraging and task engagement, particularly when transitioning from a lower-concentration feeder. Accordingly, we used 50% sucrose to maintain continuity with previous field-based learning studies^21,22^, to facilitate reliable recruitment of bees to the experimental setup and to maintain a high appetitive motivation for the bees to voluntarily participate in the extensive learning tasks which usually lasted between six and eight hours per bee**.** Our data suggest that this higher concentration did not mask individual differences in learning performance. Despite the use of 50% sucrose, we still observed substantial variation in task accuracy across individuals, with some bees performing near chance level. Furthermore, most bees (*n* = 24 out of 29) responding to 50% sucrose also responded to lower concentrations (30% and 10%) in the sucrose responsiveness assay, indicating high overall sucrose sensitivity. These findings suggest that even intermediate concentrations would have been perceived as highly rewarding and would thus unlikely yield qualitatively different results.

Importantly, we compared the obtained GRS values with those of naïve bees randomly collected from an artificial feeder. Thus, we determined whether long visual conditioning procedures (between six and eight hours per bee) altered the bees’ original evaluation of sucrose^29–32,35^. Although this procedure has the disadvantage of measuring GRS in different sets of bees (naïve and conditioned bees), the GRS of the conditioned bees did not differ from that of naïve bees, indicating that the conditioning procedure did not alter their sucrose responsiveness. The median gustatory response score (GRS) of the bees was 6, with more than half of the individuals (*n* = 16) displaying high responsiveness to sucrose (GRS 6–7). Fewer bees showed intermediate responsiveness (GRS 4–5: *n* = 6; GRS 2–3: *n* = 2), whereas a minority had low responsiveness (GRS 0–1: *n* = 7). Consequently, the variability in GRS within our experiment was relatively low compared with that in other studies, which reported a more balanced distribution across these GRS categories^37,41,42^. This limited variability may have influenced the correlation analysis.

Mujagic and Erber^44^ studied the relationship between sucrose responsiveness measured via the PER assay in the laboratory and sucrose acceptance at an artificial feeder under free-flying conditions. Their results revealed that bees accept lower sucrose concentrations at the feeder than those required to elicit a PER response of the same bees in the laboratory. Interestingly, our own data support their results, as all experimental bees readily accepted the 20% sucrose provided at the artificial feeder before they were recruited to the experimental setup. However, some bees did not extend their proboscis when stimulated with a 30% sucrose solution during the laboratory-based sucrose responsiveness assay. These findings suggest that sucrose acceptance in the field cannot be reliably inferred from laboratory-based sucrose responsiveness evaluated via the PER assay.

Similarly, we did not find a relationship between individual bees’ learning proficiency in free-flying conditions and their responsiveness to light. However, previous studies have shown a positive relationship between sucrose responsiveness, responsiveness to light and learning proficiency in harnessed bees^37,41^. The performance of the bees in our phototaxis assay was comparable to that observed in previous experiments using the same set-up^39^, indicating that the extensive conditioning period did not significantly alter their light responsiveness. However, since this was not explicitly tested, we cannot fully rule out the possibility of such an effect.

Previous studies have shown a positive correlation between sucrose responsiveness and responsiveness to light^37,41,42^. In contrast, our results reveal no correlation between the GRS and mean walking time across different light intensities, indicating no strong link between sucrose responsiveness and responsiveness to light. Methodological differences may account for these discrepancies. First, while we used similar green light compared to earlier experiments (527 nm vs. 520 nm in^37,42^), the LED illuminance differed. In addition, the bees in our study underwent extensive free-flying conditioning before the phototaxis test, which may have influenced their sensory responsiveness. While we found no evidence that these tasks affected sucrose responsiveness, their potential impact on light responsiveness remains untested and requires further investigation.

Our experiments revealed a striking difference in the relationship between responsiveness to sucrose and light and learning in harnessed versus free-flying bees. While sucrose responsiveness predicts learning performance in harnessed bees^29–33^, it does not do so in free-flying bees, at least not when measured after the training procedure. Similarly, sucrose responsiveness of harnessed bees correlates with responsiveness to light in an arena similar to that used in our study^37,41,42^. However, no such relationship was observed in bees that had previously undergone visual learning tasks under free-flying conditions.

A possible explanation for this difference may lie in the experimental conditions imposed on the bees, particularly their ability to move freely in one case but not in the other. In laboratory learning tasks, harnessed bees are “forced” to participate, with their movement restricted. This may significantly impact their intrinsic motivation. No study has so far directly compared motivational states between restrained bees and free-flying bees. Therefore, our interpretation remains a hypothesis. Here, we use ‘appetitive motivation’ to refer to hunger- and reinforcement-linked drive^13^, whereas ‘intrinsic motivation’ denotes task engagement and persistence when disengagement is possible^47^. This distinction is conceptual rather than experimentally demonstrated, but it is consistent with frameworks that separate reinforcement-linked appetitive drive from task engagement and persistence in insect cognition^47^. One could hypothesize that in such conditions, where participation in the experiment does not consider the bees’ intrinsic motivation, behavior may be driven primarily by mechanisms such as response thresholds and appetitive motivation. In contrast, learning experiments with free-flying bees closely simulate natural foraging behavior, allowing bees to leave the experiment at any time. This suggests a high intrinsic motivation to participate in the learning task, which could also explain the high general level of sucrose responsiveness found in our study in comparison with PER studies. Importantly, a high sucrose responsiveness and robust olfactory PER learning (> 80%) are well documented under restrained conditions and thus we do not argue for an absence of motivation under restraint but propose that autonomy in free-flying contexts plausibly shifts the relative weighting of appetitive drive, response thresholds, and attention-like processes. In this free-flying context, factors such as attentional processes may play a more dominant role than individual sensory response thresholds, strongly suggesting a context-dependent contribution of attentional processes, intrinsic motivation, and response thresholds when comparing free-flying and harnessed learning experiments. Attention-like selection and state-dependent sensory modulation have been demonstrated in insects^48–54^, supporting the view that these processes are adaptive and context-dependent. Furthermore, the experimental design of our study, in which each bee was subjected to two extensive learning tasks (6–8 h of training per bee), may have introduced an element of “artificial” selection, as we were only able to analyze data from bees that completed the whole procedure. Consequently, it seems plausible that this approach selectively retains highly motivated individuals.

Indeed, recent studies comparing sensory processing in active (freely moving) versus inactive (restrained) mice have shown that behavioral states modulate sensory perception. Increased locomotion and arousal in active individuals are generally associated with more robust and accurate visual encoding^55–59^. This state-dependent modulation of sensory processing has also been shown to affect associative learning performance in mice^60^. While insects also exhibit behavioral state-dependent modulation of neuronal^48–50^ and sensory processing^51^, the link between behavioral state, sensory perception, and associative learning remain unexplored.

Interestingly, previous studies showed that transfer of learned association between contexts is not symmetric, further demonstrating the impact of the bees’ degree of freedom on behavior expression. Using olfactory conditioning—either PER conditioning in harnessed bees or operant olfactory conditioning of free-walking bees in a Y-maze—it was shown that PER-trained bees transferred their choice to the Y-maze, where they could move freely^46^. This situation is comparable to our sucrose responsiveness test followed by the light responsiveness assay. In contrast, Y-maze-trained bees did not respond with a PER to odors when subsequently harnessed. Thus, regaining freedom by transferring bees from PER conditioning in a harness to the maze may be experienced as a positive situation, allowing the bee to fully express adaptive behavior toward a CS. The reverse transition, from maze conditioning under free-flying conditions to immobilization in tubes, may potentially be experienced as a negative, stressful situation, inhibiting responses to the CS. This conclusion is further supported by experiments on the transfer of visual learning between two different contexts: a virtual reality (VR) system in which bees were harnessed and a Y-maze in which they could move freely. In this case, transfer from VR to the maze significantly improved the bees’ performance, whereas the reciprocal transfer from the Y-maze to VR produced no improvement^45^. Consistent with the olfactory study, these findings indicate that reducing opportunities for free movement when transferring from the maze to harnessed VR impairs the expression of visual learning, whereas increasing them in the reciprocal transfer enhances it.

The results obtained by comparing the sucrose responsiveness of naïve and free-flying bees with that of free-flying bees trained in extensive visual learning tasks revealed no significant difference. This absence of difference was interpreted as reassurance that the responsiveness measured in trained bees had not been altered by the learning process. However, a recent study on harnessed bees demonstrated that aversive olfactory conditioning can modify responsiveness to electric shocks of increasing voltage, as assessed through their sting extension response (SER)^61^. In this experiment, shock responsiveness was first measured, followed by olfactory aversive conditioning, during which the same bees learned to distinguish between an odorant paired with a shock and a safe odorant. As a result, they learned to extend their stings in response to the punished odorant but not to the safe odorant. Three days after conditioning, shock responsiveness was measured again in these bees, revealing a consistent and persistent decrease in responsiveness to voltages lower than those used for conditioning. Introducing an appetitive conditioning phase, where one odorant was paired with a sucrose solution and another remained non-rewarded between the two shock responsiveness measurements, did not induce such a decrease in responsiveness^61^. This finding indicates that the observed decrease was specifically related to the experience of repeated and predictable shocks of a given intensity during aversive conditioning. This finding suggests that conditioning not only generated expectations about the conditioned stimulus (CS) but also about the unconditioned stimulus (US) itself. Specifically, bees reduced their responses to voltages perceived as less relevant than experienced ones do^61^.

This experiment differs from our study in several key aspects. Our measurements were conducted on different bees, specifically free-flying foragers making repeated bouts between the experimental setup and the hive and focused on appetitive rather than aversive responsiveness. Free-flying bees arrived at the experimental setup with inherently higher sucrose responsiveness, as reflected by their high gustatory response scores (GRS), indicating that the foragers in our experiments were highly responsive to a wide range of sucrose concentrations, from the lowest to the highest. While conditioning may generate a specific expectancy about a US intensity (i.e., a sucrose concentration) experienced during training, we proposed that the regular return to the hive—where foragers are exposed to intense food demand—may reset this preference, maintaining their responsiveness at a high level. This resetting would be impossible in harnessed bees, which would therefore maintain the specific expectancy about the US intensity generated by conditioning and adjust their responsiveness to less relevant intensities accordingly. We thus conclude that the previously demonstrated correlations between responsiveness to light, responsiveness to sucrose and associative learning in studies with restrained bees^29–33,35–37,41,42^ may reflect motivational differences under restrained conditions. The absence of such correlations under free-flying conditions – where the motivational differences are minimized as the bees are free to leave the experimental set-up at any time – strongly suggests the context-dependent importance of individual response thresholds and intrinsic motivation in free-flying vs. restrained learning experiments with honey bees.

## Methods

A single nucleus colony (280 × 280 mm) was introduced into an outdoor flight cage measuring 4 × 6 m at the departmental apiary of the Universität Würzburg from March to April. The cage included artificial gravity feeders providing 20% (weight/weight) sucrose solution and water ad libitum. Freshly dried and ground pollen was also provided every day in a Petri dish. All bees used for this experiment were nectar foragers captured at the sucrose feeder. The basic experimental design involved training bees on a learning task, followed by a sequence of two stimulus-responsiveness assays, sucrose responsiveness and responsiveness to light.

### Learning tasks—general procedure

We used the same conditioning protocol as previously described^22^. Before the bees (*n* = *30*) were subjected to their respective learning tasks, each bee was individually pretrained to familiarize it with the experimental set-up. The setup consisted of a rotating screen (50 cm in diameter) mounted on a pedestal placed on a table (Fig. 1). The screen featured hangers (6 cm × 8 cm), which could be attached at various locations and used to display the visual stimuli to be learned^62^. A small landing platform at the base of each hanger was allowed to provide reinforcement during both pre-training and conditioning. Both the rotating screen and the hangers had the same gray color, which appeared achromatic to the bees. During pre-training, a bee selected at the artificial feeder was placed on the landing platform of a hanger displaying no stimuli, where it was allowed to collect a 50% sucrose solution (w/w) ad libitum. Once a bee landed by itself on the platform, it was marked with a colored spot on the thorax via a Uni posca paint marker (Mitsubishi Pencil Co., Ltd.).

To ensure regular foraging in the experimental setup, each marked bee had to land on the platform and collect the reward for at least five consecutive foraging bouts. Only one bee was conditioned at a time. Any recruited bees were captured and kept in a cage to prevent disturbance to the focal bee. The two learning tasks used for conditioning—reversal learning and negative patterning (described below)—followed the same general procedure but varied in visual stimuli, type of reinforcement and number of conditioning trials. After completing pre-training, each bee was fed ad libitum with sucrose solution before returning to its colony to unload and empty its honey stomach. After a few minutes the bee returned to the experimental set-up with high appetitive motivation.

While the bee was absent from the experimental set-up, the visual stimuli for the given learning task (see specifications below) were taped to the hangers, which were attached to the rotating screen, and a 10 µl drop of the corresponding reinforcement was placed on each landing platform. Each visual stimulus was presented twice on the rotating screen. During each conditioning trial, the bee was required to approach the screen, choose between the visual targets, land on the selected platform and consume the reward (50% sucrose solution, w/w) for a correct choice, or taste the punishment (60 mM quinine solution or plain water) for an incorrect choice. Each choice made by the bee during a trial was recorded, with one trial corresponding to a single choice. If the bee made a correct choice, it was transferred to a large Plexiglas spoon containing a small droplet of sucrose solution and moved one meter away from the screen. During this time, the screen was rotated to change the relative position of the hangers to prevent positional learning, and the sucrose solution was replaced. Then, a bee was allowed to make another choice. If the bee made an incorrect choice, it usually only probed the quinine solution and was allowed to continue making choices until the correct target was selected. We did not observe malaise effects previously reported in response to quinine ingestion^63^. Typically, bees made three to five choices per foraging bout before returning to the colony. Upon the bee’s departure, all hangers were cleaned with 50% ethanol to remove potential odor markings, hanger positions were altered, and reinforcement solutions were replenished. This procedure was repeated until the required number of conditioning trials for the respective task (see below) was completed.

Once the conditioning phase was completed, non-reinforced tests were conducted. New hangers and stimuli were introduced during these tests, and no reinforcement was provided. Each test continued until the bee completed a total of 20 choices. In the absence of reinforcement, a choice was recorded when the bee landed on or touched a landing platform or stimulus. From this data, we calculated the percentage of correct choices made by each bee during each non-reinforced test.

Half of the bees were first subjected to a reversal learning task followed by a negative patterning task, whereas the other half were subjected to the reverse sequence. A non-reinforced test was performed after each task. Following each test, the bee was allowed to collect sucrose ad libitum from hangers displaying no visual stimuli over three foraging bouts. We ensured the use of highly motivated bees, which typically returned to the experimental setup within two to five minutes after leaving. Only bees that successfully completed the conditioning and testing phases for both learning tasks were included in the statistical analysis. A schematic overview of the conditioning and test phases of both learning tasks can be found in the supplementary material (Fig. S1).

### Learning tasks—reversal learning

The reversal learning protocol involved two phases. The first phase (1^st^ phase RL) consisted of differential conditioning, where one visual stimulus (A+) was associated with reward (50% sucrose solution, w/w), whereas a second stimulus (B-) was associated with punishment (60 mM quinine solution). In the second phase (2^nd^ phase RL), the reinforcement contingencies were reversed so that the previously rewarded stimulus (A+) was associated with punishment (A−) and the previously punished stimulus (B−) was paired with a sucrose reward (B+). Both phases comprised 30 reinforced conditioning choices and each phase was followed by a non-reinforced test in which the bee was allowed to make 20 choices. The second phase began after one foraging bout (three to five choices), during which only the sucrose solution was presented without visual stimuli. Throughout both conditioning and testing, the screen consistently displayed four hangers, two with stimulus A and two with stimulus B. Reversal learning was evaluated only in individuals who had successfully learned the initial discrimination during the first phase^64–67^.

The stimuli used as A and B were colored squares cut from HKS-3N and HKS-68N cardboards (5 × 5 cm HKS-N pigment papers; Hostmann-Steinberg K + E Druckfarben, H. Schmincke and Co., Germany) that appeared yellow and greenish yellow to the human eye, respectively (see Fig. S2 in the supplementary material for spectral reflectance curves and their position in the hexagon color space). Half of the bees were initially conditioned using HKS-3N as stimulus A and HKS-68N as stimulus B, while the opposite pairing was used for the other half.

### Learning tasks—negative patterning

In negative patterning, bees must learn that two stimuli are rewarded when presented individually (C+ and D+) but not when presented together (CD-). This requires bees to recognize CD as a distinct configuration rather than simply processing it as the sum of its components, C and D^68–70^. The protocol and stimulus design stimuli were adapted and modified from the Schubert et al*.* study^70^. The conditioning phase consisted of three different blocks of trials: in two blocks, either stimulus C+ or D+ was presented alone on four hangers (absolute conditioning) and paired with 10 µl of a 50% sucrose solution. In the third block, the non-reinforced compound CD- was displayed on two hangers and paired with 10 µl of plain water, whereas the remaining two hangers displayed a different compound XY+, which was paired with 10 µl of sucrose reward (differential conditioning). Including XY+ during the CD- block was necessary to maintain the bees’ foraging motivation; in the absence of any reward, they would stop visiting the experimental set-up. Although XY+ trials were included to maintain foraging motivation, in principle, this design could create alternative associations of CD- in the context of XY+. However, the critical discrimination between CD- and its elemental components remains intact, as in the original paradigm^70^.

The trial blocks were pseudo-randomized throughout the conditioning phase, with each block lasting for one foraging bout (three to five choices). Upon the bee’s return to the hive, the stimuli were exchanged, and the next block began. In total, the conditioning phase involved 120 choices: 30 each with stimuli C+ or D+ and 60 with the compound CD- and the rewarding alternative XY+. Thus, positive and negative reinforcements were equated for C and D throughout the conditioning phase to exclude elemental solutions. Following conditioning, two non-reinforced tests of 20 choices were conducted. In these tests, either C+ or D+ was presented alongside CD- on two hangers each, without reinforcement. To maintain foraging motivation, a reinforced refreshing session was provided between tests, which lasted for one foraging bout. As the bees made at least three choices, each stimulus (C+, D+, CD− vs. XY +) was presented at least once in a pseudo-random fashion during the refreshing session.

**Fig. 4:**
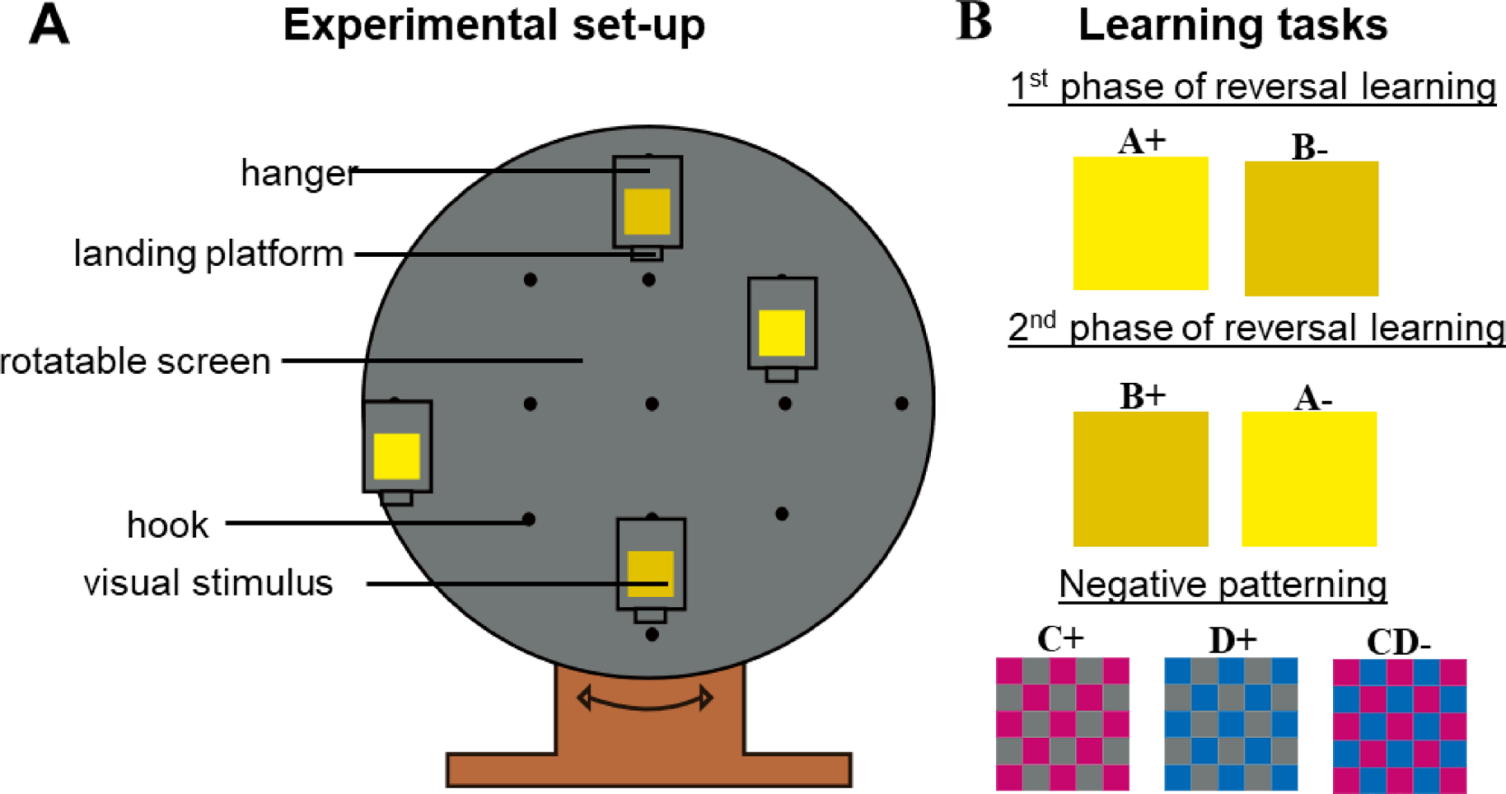
Schematic overview of (**A**) the experimental setup and (**B**) the learning tasks tested. (**A**) The rotating screen apparatus consisted of a vertical rotatable screen mounted on a stand. Hangers displaying the stimuli of the respective learning tasks (5 × 5 cm squares) were attached to the screen at various positions via hooks. Each hanger featured a small landing platform where bees could collect reinforcements during conditioning. (**B**) Overview of the learning tasks. In the first phase of reversal learning, stimulus A+ was rewarded with sucrose solution, whereas stimulus B− was punished with a bitter quinine solution. In the second phase, the reward contingencies of the first phase were reversed so that stimulus A− was punished and B+ rewarded. The stimuli used were cut from HKS-3N and HKS-68N cardboard, which appeared yellow and greenish yellow to the human eye, respectively. Half of the bees were initially trained with HKS-3N as stimulus A and HKS-68N as stimulus B, and vice versa for the other half. In the negative patterning task, bees had to learn that single stimuli C+ and D+ were each rewarded with a sucrose solution, while their compound CD- was not reinforced. The single stimuli used were checkerboard squares cut from HKS-26N cardboard (C+, pink) and HKS-44N (D+, blue) presented on a gray background, which was achromatic to the bees (HKS-92N). The compound stimulus CD- combined squares from HKS-26N and HKS-44N cardboard.

The stimuli used were checkerboard squares (5 × 5 cm) cut from different HKS-N cardboard samples (Hostmann-Steinberg K + E Druckfarben, H. Schmincke and Co., Germany). The spectral reflectance curves and their positions in the hexagon color space are provided in Fig. S2 of the supplementary material. The single stimuli consisted of 1 × 1 cm squares of either HKS-26N (C+, pink) or HKS-44N (D+, blue) on a gray background (HKS-92N). The compound stimulus CD− combined 1 × 1 cm squares of HKS-26N and HKS-44N (see Fig. 4). The rewarding alternative XY+ featured black 1 × 1 cm squares (HKS-88N) on a white copy paper background.

### Sucrose responsiveness assay

Once a bee completed the non-reinforced test for the second learning task, it was immediately captured in a small glass vial. In the laboratory, the bee was immobilized by placing the vial on crushed ice until all movements ceased (~ two to three minutes). Once immobilized, each bee was carefully harnessed in a small metal tube so that only the mouthparts could be freely moved. The head was secured with a small strip of adhesive tape, while the body remained inside the tube and was stabilized with additional adhesive tape. To facilitate bee removal after the sucrose responsiveness assay, all the adhesive tape in direct contact with the bee was coated with soft tissue.

Following fixation, the bees were fed five µl of sucrose solution (30% w/w) and placed in a dark, humid chamber at room temperature for 30 min. The responsiveness of the bees to different sucrose concentrations (0 [water], 0.1%, 0.3%, 1%, 3%, 10% and 30% w/w) was then assessed following a standard protocol^29,30,33,43,71^. The antennae were touched with a toothpick soaked in the corresponding solution, and the proboscis extension response (PER) was recorded. The assay started with water and proceeded with increasing sucrose concentrations, with a two-minute inter-trial interval to prevent sensitization effects^72^. A gustatory response score (GRS^18^) was determined for each bee by summing the number of proboscis extensions to water and the six different sucrose concentrations. The GRS could consequently range between “0” (no responsiveness) and “7” (high responsiveness). After the assay was complete, each bee was carefully removed from the harness and placed in a Petri dish for the phototaxis assay.

Since the test for individual sucrose responsiveness is typically performed before learning experiments in restrained bees^29,30^, we randomly collected bees from the artificial gravity feeder at two time points (the beginning and the end of the experiment) and measured their GRS to evaluate whether participation in the learning tasks altered their sucrose responsiveness.

### Phototaxis assay

Phototaxis, or responsiveness to light, was assessed immediately after the sucrose responsiveness assay. The bees were first placed in a Petri dish (85 mm in diameter) in constant darkness for 15 min to induce dark adaptation. The phototaxis assay was conducted according to a standardized protocol^37–42^, using the same setup described by Schilcher et al*.*^39^. After dark adaptation, each bee was introduced into an arena (35 cm in diameter) under darkness to quantify locomotor activity. The bee’s mean velocity during a two-minute period was recorded as it walked freely in the arena.

Then, 12 green light-emitting diodes (LEDs) with a wavelength of 527 nm and varying relative light intensities (3.125%, 6.2 5%, 12.5%, 25%, 50% and 100% intensity; maximum light intensity: 2.61 × 10^14^ photons/cm^2^) were successively switched on in ascending order, starting with the lowest intensity. Two LEDs of the same intensity were positioned on opposite sides of the arena. When an LED was switched on, the time required for the bee to walk was recorded. When a bee reached the LED, the light was switched off, and the opposite LED was switched on. This procedure ensured that the bees had to walk a minimum of 35 cm to reach the opposite light source. Each light intensity was tested four times per bee.

The behavior of the bees was monitored and recorded via an infrared camera mounted on top of the arena and connected to a computer, while the walking time was measured via a computer stopwatch. The mean walking time for each bee was calculated from the four trials per light intensity. Locomotor activity was analyzed with UMA tracker software^73^, which measures the distance walked in one minute. From this, the mean velocity (m/s) was calculated. Bees who did not walk in the arena within five minutes were excluded from the analysis (*n* = 6 out of 30).

### Statistical analysis

Individual consistency across performances in the learning tasks, measured by the percentage of correct choices made in the non-reinforced tests, was assessed using Spearman rank correlations. For analyses involving the second phase of reversal learning, only bees that had successfully learned the initial discrimination task in the first phase were included. This restriction is important because reversing a previously learned association requires successful learning of the initial task^67^. Among the 30 bees, 26 met this criterion. A threshold of ≥ 60% correct choices was set to classify a bee as a learner in the first phase of reversal learning^22^. For negative patterning where we had to conduct two non-reinforced tests after conditioning, we calculated a mean test performance to obtain a single measure of learning proficiency for negative patterning per bee. The two non-reinforced tests of negative patterning did not differ significantly (C+ vs. CD-: median 67.5% correct choices and D+ vs. CD-: median 70.0% correct choices; Mann–Whitney U test, *n* = *30, U* = *447, p* = *0.968*).

Sucrose responsiveness was analyzed using the GRS, calculated as the sum of proboscis extension responses across increasing sucrose concentrations. To test whether PER probability increased with increasing sucrose concentration we used a Friedman test for repeated measures, with sucrose concentration as the within-subject factor.

To assess whether the order in which the learning tasks were conducted or the rewarding color used in reversal learning (*group_RL*) influenced performance in the three learning tasks, generalized linear mixed models (GLMMs) were applied. These models had a binomial error structure with a logit-link function and used the percentage of correct choices in each non-reinforced test as the dependent variable. Task order and rewarding color were included as fixed factors, whereas bee identity was a random factor. Additionally, we performed additional GLMMs to assess whether responsiveness to sucrose and light influenced performance in the three learning tasks. Therefore, we used the percentage of correct choices in each non-reinforced tests as dependent variable and GRS and mean walking time (mean_WT) in the phototaxis assay as fixed factors. Bee identity and the trial of the phototaxis assay (1–6, PT_trial) were included as random factors. Model selection followed a stepwise approach, starting with the most complex model and gradually removing factors. The models were compared using an ANOVA, and the best fit was determined based on the lowest Akaike information criterion (AIC) value^74,75^.

Spearman rank correlations were used to evaluate the relationships between individual test performance in reversal learning and negative patterning, locomotor activity, and responsiveness to light and sucrose. Principal component analysis (PCA) was conducted on the six phototaxis values of each bee to reduce dimensionality and identify latent structures. This statistical method transforms the original six phototactic values per bee into a new set of variables called principal components. The first principal component (PC1), which accounted for the largest proportion of variance in the data, was interpreted as a latent dimension representing overall phototactic responsiveness. Component scores from PC1 were computed for each individual bee, providing a single composite metric summarizing its overall phototactic tendency across all trial types referred to as “phototaxis scores” (SPSS, IBM). These phototaxis scores were used as predictor variables in subsequent analyses. Gustatory response scores (GRS) of the tested bees were compared with those of control bees caught at the artificial feeder using Mann–Whitney U tests to determine whether participation in the learning tasks affected sucrose responsiveness compared with that of naïve bees.

GLMMs were constructed via R statistical software^76^ with the package lme4^77^. All other statistical analyses and graphs were generated via GraphPad Prism version 9.4.0 (GraphPad Software Inc., San Diego, California, USA). The significance level was set at α < 0.05 for all tests.

## Supplementary Information

Below is the link to the electronic supplementary material.

Supplementary Material 1

## Data Availability

The data is available via the following DOI: 10.6084/m9.figshare.29493188.v3
