# Supplementary material for: Visual learning performance in free-flying honey bees is independent of sucrose and light responsiveness and depends on training context
Source: Sci Rep. 2026 Jan 9;16:1319. doi: 10.1038/s41598-025-34900-9 (PMC12796264; doi:10.1038/s41598-025-34900-9)
Supplement: Supplementary file 1 — Supplementary Material 1 [file 41598_2025_34900_MOESM1_ESM.docx]

## **Supplementary material**

## **Visual learning performance in free-flying honey bees is independent of sucrose and light responsiveness and depends on training context**

## Valerie Kuklovsky^1*^, Aurore Avarguès-Weber^2, ¥^, Martin Giurfa^3,4, ¥^, and Ricarda Scheiner^1, ¥^


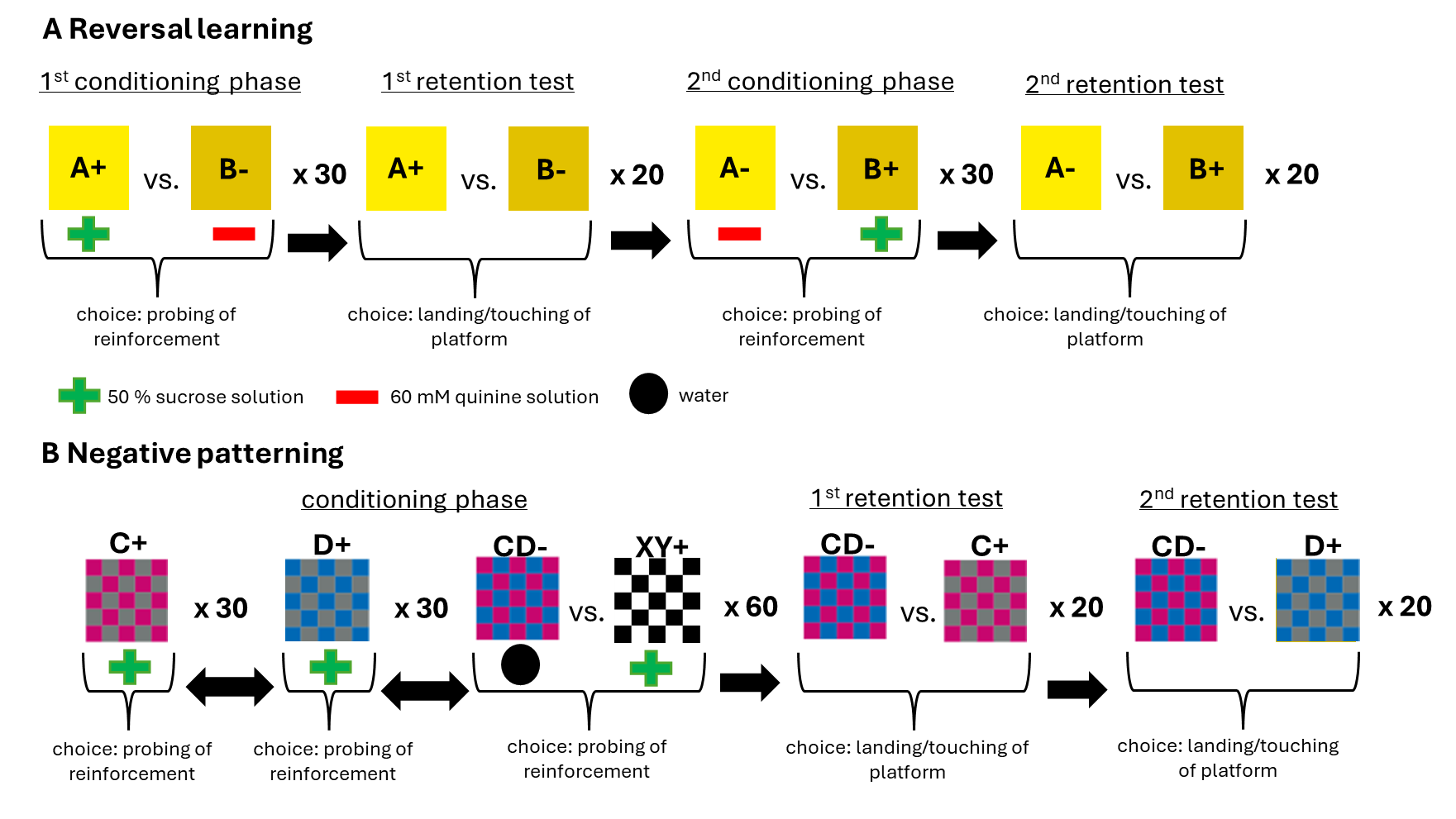


***Fig. S1. Schematic overview of conditioning and non‑reinforced retention tests for A) reversal learning and B) negative patterning****.* ***A)*** *The reversal learning task consisted of two phases, each comprising a conditioning phase followed by a non‑reinforced retention test. In the first phase, stimulus A+ was paired with sucrose reward and stimulus B- with quinine punishment (see Fig. 4). During 30 conditioning choices, bees selected between A+ and B- presented simultaneously on the rotating screen. A correct choice was scored when the bee landed on A+ and consumed the sucrose solution; an incorrect choice was scored when the bee landed on B- and contacted the quinine solution. Stimulus positions were rotated between choices. After completion of the 30 choices, a non‑reinforced retention test followed in which A+ and B- were presented without reinforcement, and 20 choices were recorded. In the second phase, reinforcement contingencies were reversed (A-, B+) and bees were trained over 30 choices, followed by a second non‑reinforced retention test of 20 choices.* ***B)*** *The negative patterning task comprised 120 conditioning choices. Bees experienced 30 choices each with C+ or D+ alone (rewarded with sucrose), and 60 choices with the compound CD- (not reinforced, water) presented alongside a rewarding alternative XY+. Correct choices were scored when bees landed on C+ or D+ and consumed the sucrose solution; incorrect choices were scored when they landed on CD- and contacted water. After completion of 120 conditioning choices, two non‑reinforced retention tests were conducted, each presenting either C+ or D+ together with CD- for 20 choices.*

***Fig. S2 Spectral properties of the colored stimuli used in reversal learning and negative patterning.*** *The spectral curves were measured with a spectrophotometer (Avantes AvaSpec-ULS2048L). The reflectance curves for the HKS paper stimuli were measured with the Avalight Xenon source and FCR-7UV200-2-M2 optical fiber. The hexagon color space is a model representing color representation from photoreceptor excitation based on generalized color opponency. Perceptual discrimination between colors could be quantified via the Euclidean distance between colored stimuli*^1^*. For the calculations of the hexagon distances of the stimuli, the spectral sensitivities of the honey bee photoreceptor*^2^*, the standard daylight function D65*^3^ *and the gray background of the hangers were used.* ***A)*** *Spectral reflectance curves of the yellow (3N) and greenish yellow (68N) stimuli used in reversal learning and of the pink (26N), blue (44N), black (88N) and gray (92) colors used to create the stimuli in negative patterning****. B)*** *Loci of the colored stimuli in a hexagon color space for the trichromatic color vision of honey bees. The distances of the stimuli used in reversal learning (3N and 68N) were 0.07 hexagon units and 0.07 for the colors used in negative patterning (26N and 44N). The mean distance between all stimuli used in experiment 1 was 0.30 ± 0.04 hexagon units. Taken from the supplementary material of* ^4^.

**Fig. S3 A)** Percentages of tested bees (n = 29) showing proboscis extension responses upon stimulation with water and different sucrose concentrations. **B)** Boxplots of the gustatory response scores (GRS) of the tested bees (n = 29) which were subjected to the learning tasks and bees which were not subjected to any learning task and collected from the artificial gravity feeder at the start (control 1, n = 19) and end (control 2, n = 30) of the experiment. The black lines in the boxplots depict the median GRS of the different groups. The median GRS of the tested bees did not significantly differ from those of the control bees 1 and 2 (Mann-Whitney U test; control 1: n = 19, U = 189, p = 0.06; control 2: n = 30, U = 374, p = 0.35).

**Fig. S4 Mean walking times (s) of the bees for the different relative light intensities during the phototaxis assay.** The mean walking times decreased with increasing relative light intensities. We found a significant negative correlation between mean walking times and the relative light intensity (Spearman rank correlation; n = 24, rho = -0.35, p < 0.0001). The bees walked faster towards a colored light stimulus of higher intensity. Means and standard errors (S.E.M) are shown.

**Fig. S5 Relationship between the general locomotor activity of the bees and their test performances in reversal learning and negative patterning.** The locomotor activity was assessed via the mean velocity (in m/s) of the bees during 2-minute dark walks in the phototaxis arena. The test performances were measured as percentage of correct choices made in the non-reinforced test during 20 trials. The GRS was not significantly correlated with the test performances in **A)** the first phase of reversal learning (1^st^ phase RL: Spearman rank correlation; n = 29, rho = 0.08, p = 0.69, R^2^ = 0.01 ), **B)** the second phase of reversal learning (2^nd^ phase RL: n = 29, rho = 0.02, p = 0.92,, R^2^ = 0.001) and **C)** negative patterning (NP: n = 29, rho = 0.114, p = 0.49, R^2^ = 0.06). Each dot represents the data of a single bee. The lines indicate a linear regression with the 95 % confidence intervals shown as grey dotted lines. Red dashed lines represent non-significant correlations.

**Table S1. GLMM analysis of the bees’ performances in the non-reinforced tests of the 1st phase of reversal learning.** The model with the best fit is highlighted in bold. The p-value indicates the comparison of the concerning model with the model including one level of higher complexity.

| **Models** | **df** | **AIC** | **Log-Lik** | **χ^2^** | **p(>χ^2^)** |
| --- | --- | --- | --- | --- | --- |
| model1: response ~ order * group_RL + (1 \| subject) | 5 | 538.70 | -264.35 | - | - |
| model2: response ~ order + group_RL + (1 \| subject) | 4 | 536.74 | -264.37 | 0.04 | 0.84 |
| model3: response ~ order + (1 \| subject) | 3 | 534.85 | -264.42 | 0.11 | 0.75 |
| **model4: response ~ (1 \| subject)** | **2** | **533.73** | **-264.87** | **0.89** | **0.35** |

**Table S2. GLMM analysis of the bees’ performances in the non-reinforced tests of the 2nd phase of reversal learning.** The model with the best fit is highlighted in bold. The p-value indicates the comparison of the concerning model with the model including one level of higher complexity.

| **Models** | **df** | **AIC** | **Log-Lik** | **χ^2^** | **p(>χ^2^)** |
| --- | --- | --- | --- | --- | --- |
| model1: response ~ order * group_RL + (1 \| subject) | 5 | 626.80 | -308.40 | - | - |
| model2: response ~ order + group_RL + (1 \| subject) | 4 | 625.39 | -308.69 | 0.59 | 0.44 |
| model3: response ~ order + (1 \| subject) | 3 | 626.32 | -310.16 | 2.9 | 0.09 |
| **model4: response ~ (1 \| subject)** | **2** | **625.39** | **-310.70** | **1.07** | **0.3** |

**Table S3. GLMM analysis of the bees’ performances in the non-reinforced tests of negative patterning.** The model with the best fit is highlighted in bold. The p-value indicates the comparison of the concerning model with the model including one level of higher complexity.

| **Models** | **df** | **AIC** | **Log-Lik** | **χ^2^** | **p(>χ^2^)** |
| --- | --- | --- | --- | --- | --- |
| model1: response ~ order * group_RL + (1 \| subject) | 5 | 1158.1 | -574.05 |  | - |
| model2: response ~ order + group_RL + (1 \| subject) | 4 | 1156.1 | -574.05 | 0.004 | 0.95 |
| model3: response ~ order + (1 \| subject) | 3 | 1154.4 | -574.19 | 0.28 | 0.59 |
| **model4: response ~ (1 \| subject)** | **2** | **1152.7** | **-574.33** | **0.29** | **0.59** |

**References**

1. Chittka, L. The colour hexagon: a chromaticity diagram based on photoreceptor excitations as a generalized representation of colour opponency. *Journal of Comparative Physiology A* **170**, 533–543 (1992).

2. Peitsch, D. *et al.* The spectral input systems of hymenopteran insects and their receptor-based colour vision. *Journal of Comparative Physiology A* **170**, 23–40 (1992).

3. Judd, D. B. *et al.* Spectral distribution of typical daylight as a function of correlated color temperature. *J Opt Soc Am* **54**, 1031–1040 (1964).

4. Finke, V., Scheiner, R., Giurfa, M. & Avarguès-Weber, A. Individual consistency in the learning abilities of honey bees: cognitive specialization within sensory and reinforcement modalities. *Anim Cogn* **26**, 909–928 (2023).
